# Drought assessment has been outpaced by climate change: empirical arguments for a paradigm shift

**DOI:** 10.1038/s41467-022-30316-5

**Published:** 2022-05-17

**Authors:** Zachary H. Hoylman, R. Kyle Bocinsky, Kelsey G. Jencso

**Affiliations:** 1 Montana Climate Office, W.A. Franke College of Forestry and Conservation, University of Montana, 32 Campus Dr., Missoula, MT 59812 USA; 2 Department of Forest Management, W.A. Franke College of Forestry and Conservation, University of Montana, 32 Campus Dr., Missoula, MT 59812 USA; 3 Crow Canyon Archaeological Center, Cortez, CO 81321 USA

**Keywords:** Environmental impact, Hydrology, Climate-change impacts

## Abstract

Despite the acceleration of climate change, erroneous assumptions of climate stationarity are still inculcated in the management of water resources in the United States (US). The US system for drought detection, which triggers billions of dollars in emergency resources, adheres to this assumption with preference towards 60-year (or longer) record lengths for drought characterization. Using observed data from 1,934 Global Historical Climate Network (GHCN) sites across the US, we show that conclusions based on long climate records can substantially bias assessment of drought severity. Bias emerges by assuming that conditions from the early and mid 20th century are as likely to occur in today’s climate. Numerical simulations reveal that drought assessment error is relatively low with limited climatology lengths (~30 year) and that error increases with longer record lengths where climate is changing rapidly. We assert that non-stationarity in climate must be accounted for in contemporary assessments to more accurately portray present drought risk.

## Abstract (web summary)

Climate has changed over the last century, yet this change is seldom accounted for in drought assessment. This study quantifies drought bias due to climate change and suggests adjustment to align monitoring with contemporary risk.

## Introduction

Accurately monitoring water availability is critical for sustainable water resource management as Earth’s climate continues to change and drought impacts human populations^1,2^, food production^3^, and valuable ecosystem services^4–6^. In the United States (US) drought monitoring ensures land managers, practitioners and producers have local and regional information to prepare for and respond to water scarcity^7^. This monitoring is conducted across scales; spatially from local to regional scales, temporally from the evolution of flash droughts^8^ to long-term deficits in surface water supplies, and institutionally from local, state, and national organizations. Drought monitoring at the national scale is led by the National Drought Mitigation Center (NDMC), the United States Department of Agriculture (USDA), and the National Oceanic and Atmospheric Administration (NOAA), who produce weekly drought maps constituting the United States Drought Monitor (USDM)^9^. The USDM is generated using a synthesis of many datasets by experts and input from a variety of entities (including state drought task forces) that rely on the convergence of evidence to define drought severity; from D0 (“abnormally dry”) to D4 (“exceptional drought”). These drought categories trigger critical emergency services and the distribution of several US federal disaster assistance programs related to the livestock and agricultural industry (e.g. the Livestock Forage Disaster Program, the Emergency Assistance for Livestock, Honeybees and Farm Raised Fish Program and the Emergency Haying & Grazing – Conservation Reserve Program). Disaster relief programs tied to the USDM provide hundreds of millions to billions of dollars per year in financial disaster assistance^10^.

Weekly drought assessments reflect how current conditions compare to a historical record of events and are critical for understanding the degree of moisture surplus or deficit in a particular location. Variables representing the state of water availability—e.g. precipitation, evapotranspiration, soil moisture, and streamflow—are used to evaluate conditions over varying timescales, which in turn represent different forms of drought. These indicators are then compared to impact and condition monitoring reports which are used to validate physical drought assessments. There are many drought metrics in use today^11^, most of which rely on a statistical framework that standardizes raw values into anomalies using probability statistics^12–14^. One of the most widely recommended drought metrics for operational use is the Standardized Precipitation Index (SPI)^12,15,16^, which estimates the probability of observing a certain magnitude of rainfall over a timescale of interest, customarily ranging from weeks to years.

The need to standardize an approach for computing drought metrics led to several studies that establish “best practices” for their computations^12,17–20^. A key consideration is the climatology length, or the time period used as a reference frame. The apparent severity of any given drought event depends on this frame of reference ^21^, especially within the context of climate change. Oft-cited studies in drought monitoring argue uncertainty declines with longer climatological records^12,17,22^, with approximately 60 years (or more) of observations considered requisite to promote parameter stability in drought models. This conclusion is currently included in drought monitoring recommendations by the World Meteorological Organization^23^. In contrast to drought monitoring, weather and climate sciences typically use climate normals based on 30 years of data that are updated every decade to describe average conditions and associated anomalies. These climate normals are intended to account for, at least in part, climate variability and change^24^. Updating expected climatic conditions (similar to climate normals) is critical in a non-stationary system to accurately contextualize conditions. If this concept is ignored, climate change may render current reference frames inappropriate for operational water resource assessment^21^.

**Fig. 1:**
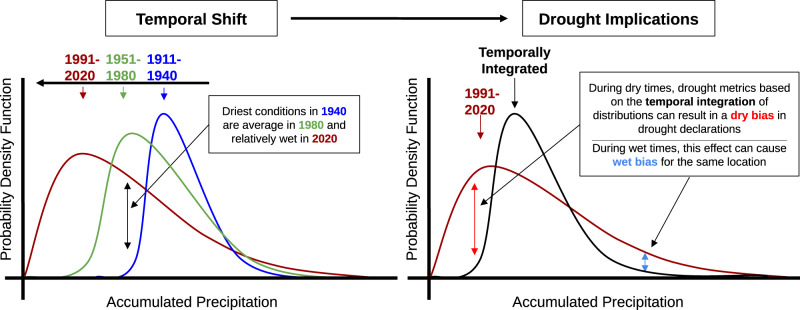
Conceptual model describing the drought metric bias associated with a non-stationary climate scenario. A theoretical accumulated precipitation dataset is presented on the horizontal axis, while the associated probability density function (PDF) is on the vertical axis. [left] Conceptual model showing one way that probability distributions can shift in time when conditions transition from a wetter, less variable state to a drier, more variable state. [right] Demonstration of how this shift can produce both a dry bias during dry times and a wet bias during wet times for a theoretical distribution.

Despite the research establishing drought metric best practices, the impact of non-stationarity in climate systems, especially those driven by anthropogenic climate change, are not currently accounted for in conventional drought metrics and drought monitoring products such as the USDM. Here, we demonstrate that this omission results in increased drought metric error and bias where climate has shifted substantially from the time-integrated period-of-record distribution (Fig. 1). If climate change is not accounted for in drought assessment, “drought” conditions (or water surplus) may become commonplace. However, is a “drought” really occurring if aridity is the “new normal”?

## Results

### Conventional methods ignore climate change

**Fig. 2:**
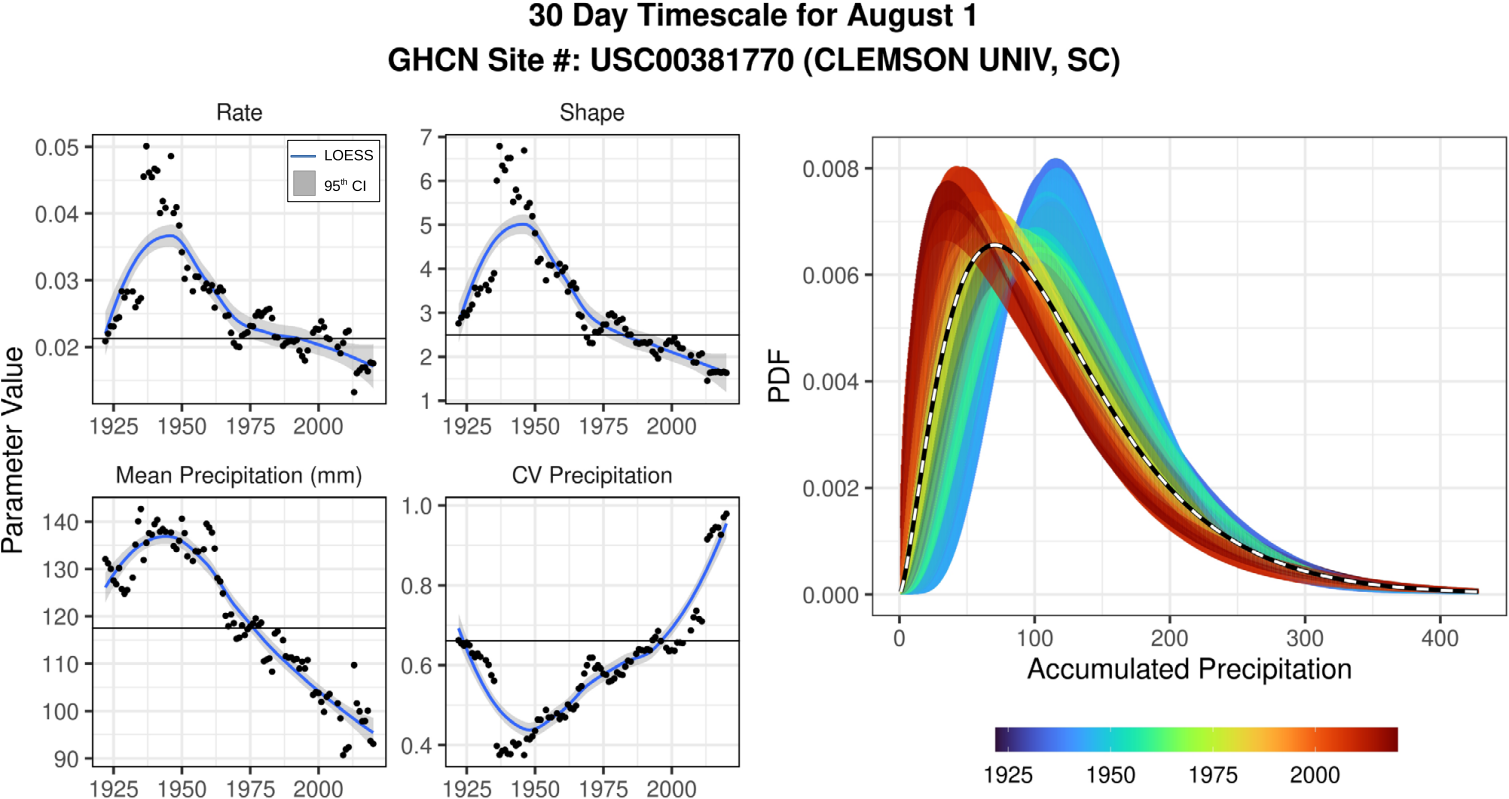
Probability distribution shift for Global Historical Climatology Network (GHCN) site USC00381770 located at Clemson University in South Carolina. [left] Subplots show 30-year moving window values of the gamma distribution rate and shape parameters, mean precipitation and coefficient of variation (CV) of precipitation for a 30-day timescale on August 1st. Horizontal lines represent values computed using the temporally integrated distribution. [right] Probability density functions (PDFs) for each of the 30-year moving windows. The color scale represents the 30-year moving window’s final year and the black and white dashed line represents the temporally integrated PDF.

The conclusions of previous research investigating the influence of climatology length on drought assessment uncertainty were based on a core assumption; the probability distribution that describes meteorology across the period of record is unchanging. In other words, the sampled data comes from a distribution that is stationary in time, “fluctuat[ing] within an unchanging envelope of variability”^21^. Under this assumption, the incorporation of “extreme events” from several decades earlier is thought to better represent the true distribution. However, precipitation dynamics, and the probability density functions (PDFs) that describe them, are indeed changing in time (for example: Fig. 2, S7, S8). Importantly, the change in precipitation dynamics is non-random, nonlinear, influenced by anthropogenic climate change^25^ and contributing to bias in our assessment of contemporary drought across the US. In this analysis, we focus on precipitation dynamics, but it is important to highlight that the concepts presented here are applicable to any analysis that utilizes a historical reference frame to standardize raw values (e.g. temperature, evapotranspiration, streamflow, snowpack, etc.) under non-stationary conditions.

Milly and colleagues^21^ evaluated the use of PDFs in water resource assessments and concluded that stationarity assumptions are inappropriate in the context of climate change. In contrast to the longest climatology (the “period-of-record”), the evaluation of *contemporary likelihood* based on a recent reference frame (current climatic conditions) puts events that impact planners, managers, and producers *today* into a relevant context — this is the objective of modern drought monitoring. In fact, research suggests that communities and producers rely heavily on near-term memory and recent experiences for decision making^26^, behavior reflective of adaptation to non-stationary environments. Here, we demonstrate drought conditions defined using period-of-record climatologies are biased by historical conditions that are assumed to have a higher probability of occurrence than has recently been observed, due to climate change. These findings warrant a discussion of conventional drought monitoring practices and how the drought management community may bring them into alignment with contemporary drought risk.

### Deriving normals in the context of climate change

**Fig. 3:**
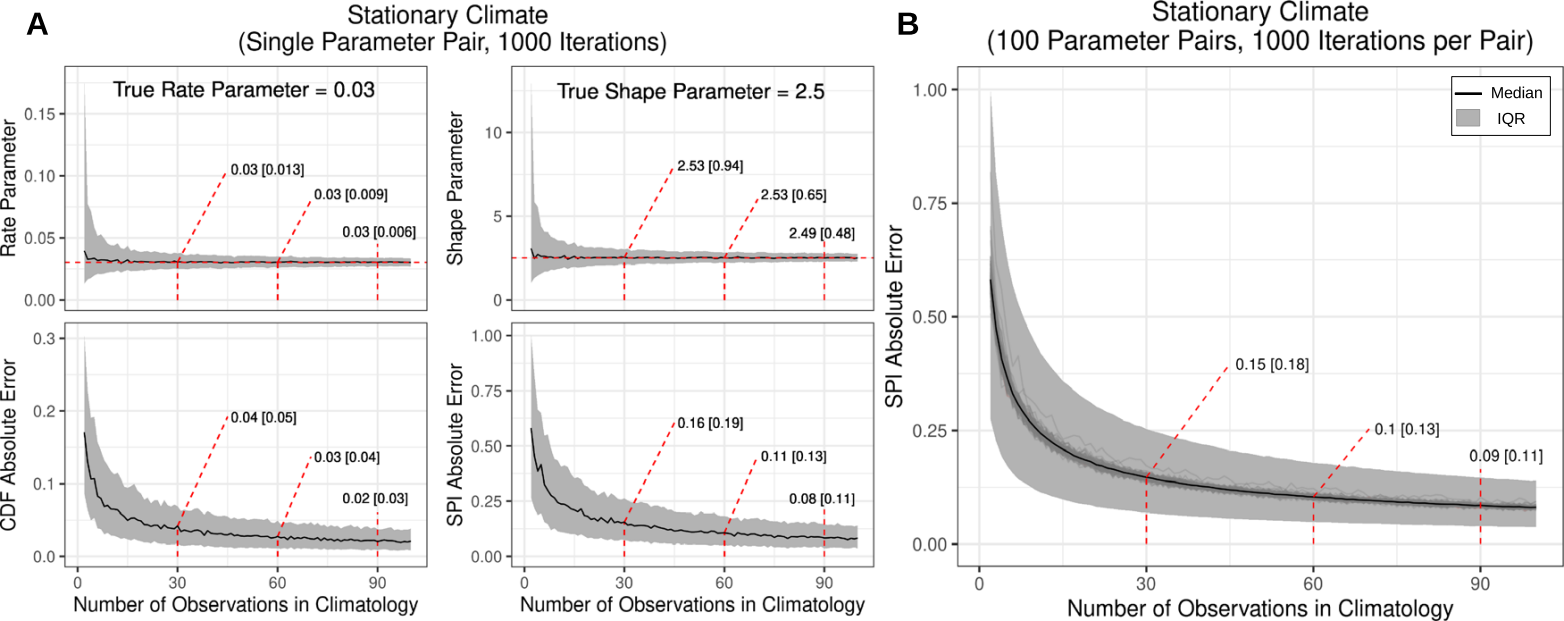
Summary of simulations that fit gamma probability distributions to a known stationary distribution. These simulations use random samples of varying climatology lengths (from 2 to 100) and the L-moment method for estimating gamma parameters. **A** Subplots showing the detailed simulation results for a single gamma distribution pair derived from 1000 simulations; rate and shape parameters as well as the cumulative distribution function (CDF) absolute error and Standardized Precipitation Index (SPI) absolute error are presented. Black lines and gray ribbons represent the median [interquartile] value of the 1000 simulations. **B** Replication of the aforementioned simulation for 100 randomly selected gamma distribution parameter pairs, each simulated 1000 times, focused on the absolute SPI error.

**Fig. 4:**
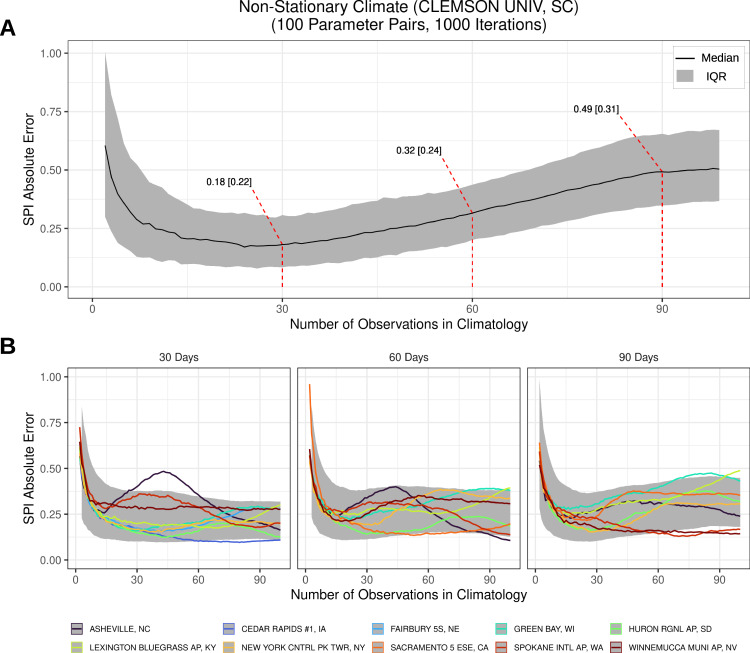
Summary of simulation results for a non-stationary distribution using the observed 30-year moving window gamma parameters from 11 Global Historical Climatology Network (GHCN) sites. The Standardized Precipitation Index (SPI) was estimated by fitting a gamma distribution to random samples of differing lengths, with each random sample being generated by the observed moving window generative gamma distribution for each site. The absolute SPI error was then computed using the known SPI value from the known (observed 30-year moving window) distribution for the most recent observation (e.g. 2020, consistent with operational SPI calculations). **A** Detailed simulation results for GHCN site USC00381770 located at Clemson University in South Carolina for the 30-day SPI for August 1st. Black lines and gray ribbons represent the median [interquartile] value of the 1000 simulations. **B** Results for 10 additional GHCN sites for various geographical locations and 30-, 60- and 90-day timescales for August 1st. Gray shading represents the interquartile range for simulations at all sites for each timescale.

Our numerical simulations indicate there are substantial differences in absolute SPI error depending on whether precipitation datasets are derived from a single probability distribution (simulating a stationary climate; Fig. 3) or from many probability distributions that vary as a function of time (simulating a non-stationary climate; Figs. 2, 4). Our stationary climate simulations confirm L-moment-based methods for fitting two-parameter gamma distributions are effective at estimating the generating gamma distribution given a relatively low number of observations (Fig. 3a). Uncertainty in the gamma distribution parameter estimates (interquartile range [IQR] ribbons about Fig. 3a rate and shape parameter plots) declined sharply as the number of observations in the climatology increased from 2 to 30 observations. Over 1000 simulations, parameter estimates converged on a median [IQR] rate parameter estimate of 0.03 [0.013] and a shape parameter estimate of 2.53 [0.94] with only 30 observations; the true rate and shape parameter pair were 0.03 and 2.5, respectively (Fig. 3a). This reduction in parameter estimate uncertainty was associated with a sharp decline in the absolute cumulative distribution function (CDF) error (i.e. the discrepancy between the observed and modeled distribution) and SPI error as observations increased from 2 to 30 observations (Fig. 3a). A slight decrease in uncertainty of parameter estimates occurred when 60 (0.03 [0.009] and 2.53 [0.65]), or 90 (0.03 [0.006] and 2.49 [0.48]) observations were included. Ultimately, the absolute SPI error was 0.16 [0.19], 0.11 [0.13] and 0.08 [0.11] for 30, 60 and 90 observations respectively. Absolute SPI error estimates exhibited little variation when simulations were conducted across observed model parameters associated with 30-, 60- and 90-day timescales (Figs. S1, 3b) indicating that this error analysis is generally representative of common operational SPI calculations.

The non-stationary simulations using observed 30-year moving windows from Global Historical Climatology Network (GHCN) stations confirm that contemporary drought metric error (computed with respect to the current 30-year distribution) does not necessarily decrease given a longer climatology in locations where climate change velocities are high (Fig. 4). For example, at GHCN site USC00381770 located at Clemson University, South Carolina (Fig. 2), the absolute SPI error initially declined in a similar fashion to the stationary climate simulations to achieve an absolute error of 0.18 [0.22] with 30 observations (Fig. 4a). However, error more than doubled as the number of observations increased from 30 to 90 samples (SPI error = 0.49 [0.31] when 90 observations were included). In some cases, error was minimized with less than 30 observations (e.g. at Spokane International Airport, WA, and Asheville, NC for 30-day SPI) or greater than 30 observations (e.g. at Huron Regional Airport, SD and Cedar Rapids #1, IA for 30-day SPI). This analysis across sites indicates the relationship between the absolute SPI error and the number of observations in the climatology is site and timescale specific and rarely follows the error-climatology length relationship consistent with a stationary climate (Fig. 4b). This important result contradicts the conventional wisdom that error decreases with increasing climatology lengths when computing error based on contemporary probabilities.

Our results demonstrate that there is a balance that must be struck to best account for non-stationarity in drought assessment. On one hand, there is a fundamental relationship between the number of observations available and the uncertainty associated with parameterization for any statistical distribution, stationary or otherwise (Fig. 3). On the other hand, error does not necessarily decrease as climatology length increases in a non-stationary climate (Fig. 4). In fact, the “true” distribution in a non-stationary time series is latent to the observer, who, without prediction, is fated to assess normalcy based solely on past, and therefore outdated, conditions. Therefore, error may increase substantially depending on the location-specific climate change velocity^27^ (Figs. 2, 4). In monotonic non-stationary scenarios, a time-integrated distribution (for example 90 observation climatology) will be diluted by increasingly outdated and progressively less probable observations and cause the integrated distribution to drift farther from the contemporary distribution (Fig. 1). It is important to emphasize that this is a site-specific phenomena; locations that have experienced little or no change (i.e. stationary climates) will have less SPI error with increasing climatology lengths—but in practice, this stationary climate example is the exception, not the rule (Fig. S2).

### Drought metric bias and climate change

**Fig. 5:**
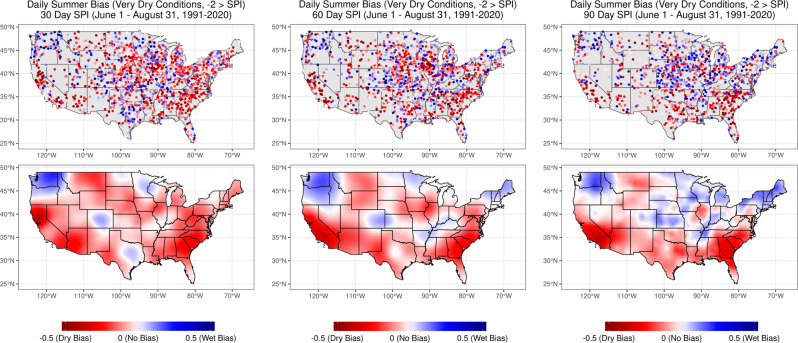
Standardized Precipitation Index (SPI) bias for Global Historical Climatology Network (GHCN) sites across the United States during periods with SPI < −2 (very dry conditions) defined using the period-of-record SPI timeseries. Bias was computed as the median daily difference between the period-of-record SPI and the 30-year (“contemporary”) SPI from June 1 to August 31, 1991–2020. Dry bias (represented by red) denotes locations where the period-of-record reports conditions that are drier than the most recent 30 years for [left] 30-day, [middle] 60-day and [right] 90-day timescales.

Non-stationary precipitation probability distributions have substantial impacts on drought metric computation and, ultimately, drought metric bias when current methodologies are followed. We observed large differences between SPI values across the US when drought metrics were computed using the longest possible, time-integrated, period-of-record climatology (greater than 70 years, representing established best practices) versus the most recent 30-year climatology (Fig. S2). Our results also indicate that these differences vary substantially across timescale and moisture state (Fig. 5, S4, S5, S6). Generally, spatial patterns of bias (dry versus wet) are consistent across timescales when controlling for moisture states; however, the magnitudes of bias vary. Drought metric bias was exacerbated as the dryness or wetness state increased with respect to the period-of-record climatology (Fig. 5, S3, S4, S5, S6).

“Hot spots” of dry bias—i.e. where the period-of-record climatology suggests conditions are drier than the 30-year climatology—were strongly apparent in the southeastern and southwestern US when evaluating the average bias across all days and wetness/dryness states (Fig. S2). Dry biases were more spatially extensive across the United States (i.e. not constrained to the southwestern and southeastern US) when the period-of-record SPI time series were less than −2 (Fig. 5), and therefore categorized as “exceptional” or “D4” drought in the USDM classification^28^. This bias was especially apparent when SPI was computed using a 30-day timescale (Fig. 5). During these historical periods of drought, there was a dry bias for 65.1%, 61.2%, and 56.5% of stations analyzed, for 30-, 60- and 90-day timescales, respectively. Overall, the southwestern and southeastern portions of the US were associated with the most consistent dry bias and exhibit the greatest magnitude of bias. Conversely, a large portion of the eastern US exhibits a strong wet bias—i.e. where the period-of-record climatology suggests present conditions are wetter than the 30-year climatology—along with the Midwest and Pacific Northwest regions (Fig. S2). This wet bias was accentuated during extremely wet time periods (when period-of-record SPI values are greater than 2) where we observed a consistent and large wet bias across the US (Fig. S3). This wet bias was consistent across timescales, even at the station scale; there was a wet bias for 93.5%, 90.6%, and 88.8% of stations, for 30-, 60- and 90-day timescales respectively.

Our analysis indicates both dry and wet bias, each of which has significant implications for either side of the drought assessment spectrum. The extensive dry bias will influence the perspective of decision makers on drought magnitude such that drought will appear *more* *severe* than is appropriate for contemporary conditions. Contrary to this result, some locations (such as the Pacific Northwest and the American Midwest) are trending wetter, and exhibit a strong wet bias during dry times. Drought in these regions will be considered *less* *severe* than is appropriate given the current climate state.

## Discussion

The National Oceanic and Atmospheric Administration’s (NOAA) recent analysis of billion-dollar weather and climate disasters reported drought as the second most costly disaster type in the US, behind only tropical cyclones^29^. Since 1980, major drought events have produced an average of $9.3 billion in inflation-adjusted damages per event, with three events exceeding $30 billion in 1980, 1988, and 2012. Damages associated with these catastrophic droughts are offset by federal disaster assistance programs related to livestock and crop risk management which have become a critical component of the U.S. agrosystem. For example, the Livestock Forage Disaster Program (LFP), which is triggered by the USDM drought severity categories, provided over 2.5 billion dollars of disaster relief in 2012^10^. As important as disaster relief is for producers to promote climate resiliency, we assert that drought monitoring and associated risk management programs need to be aligned with contemporary drought risk, else a divergence in actual risk and disaster response may occur.

The concepts responsible for the non-stationary bias reported in this study are not only applicable to the SPI, but also to any metric that uses a historical reference period to characterize anomalies. Multivariate drought metrics such as the Standardized Precipitation Evapotranspiration Index (SPEI)^13^ rely on the same general probabilistic framework and are susceptible to non-stationary bias. Climate change impacts on temperature^30^ and atmospheric aridity (e.g. vapor pressure deficit^31^) are generally more pronounced and unidirectional than precipitation^30^ which may cause a more consistent bias in alternative metrics. Locations with temporally co-occurring reductions in precipitation and increases in evaporative demand will be the most susceptible to significant multivariate drought metric bias due to climate change. Traditional parametric anomalies (z-scores) and non-parametric approaches such as empirical cumulative distribution quantiles (empirical percentiles) are also sensitive to non-stationarity bias where climatology lengths are not considered.

Our research highlights the need to produce a standardized approach to account for non-stationarity in drought monitoring so that decision makers are basing conclusions on similar datasets. Thirty-year climate normals have been used widely to acknowledge this level of climatic complexity in a simple and standardized way and are currently indoctrinated into many scientific practices^32,33^. Our analysis supports this 30-year moving window approach to balance the statistical constraints of PDF parameterization while retaining a contemporary reference frame. This methodology represents non-stationarity while leveraging the existing infrastructure for drought monitoring across the US, which is strongly dependent on traditional drought metrics such as the SPI^11^. A moving window, retrospective approach is also likely to mirror the reference frames used in decision making by agricultural producers and others most impacted by drought, and thus may be more intuitive to those communities^26^. One potential issue with a moving window approach is that the relationship between the drought metric absolute error and the number of observations in the climatology is site and timescale specific, due to differences in climate change velocities. Other more computationally intensive approaches that account for localized variability have also been proposed^34–36^. These leverage climate indices as external covariates, time-varying moments and/or Generalized Additive Models to estimate momentary generative distributions through time. These methods represent a novel approach that may be considered, but they would require a significant change in the established drought monitoring practices. Ultimately, further dialogue is needed within the drought and water resources communities on how to better characterize drought and contemporary risk in an era of rapid change.

## Methods

### Study area and time period of interest

Our study area was defined as the contiguous US, which features a diverse range of climatic conditions and has produced a wealth of long-term meteorological datasets. For this study we focus our analysis and results on the summer time period (June 1–August 31); however, data was considered back to March 4th for certain timescales and dates (e.g. 90 days prior to June 1st, discussed below). We chose this time period for two primary reasons: (1) drought conditions are common and often strongly impactful for agricultural systems during the summer months; and (2) to maximize the number of long-term datasets available for analysis. Drought metric computations are highly sensitive to missing data, especially when computation of drought metrics is based on the summation of data over a given timescale (for example 30 days) as is the case for Standardized Precipitation Index (SPI). Accordingly, we applied very restrictive constraints on whether data from a specific station or year was included in our analysis (described more below).

### Global Historical Climatology Network

We used the Global Historical Climatology Network (GHCN)^37^ as a primary dataset for our analysis. We processed the GHCN data in a stepwise process to ensure quality and completeness of data, while acknowledging that computations based on different timescales (here, 30, 60, and 90 days) require different amounts of complete data. To begin, we filtered the GHCN data for stations that started reporting in 1950 or earlier and were currently reporting as of 2020. This accomplishes two initial constraints, 1. The data record has the potential to have 70 years or more of complete data (considered the minimum number of years to compute the “period-of-record” climatology) and 2. The data from a station is considered “operational” in 2020 and therefore may be used in present-day drought monitoring. Next, we filtered the data for data completeness for different timescales. For example, a 30-day timescale requires complete data for 30 days prior to any day of interest. Here, our period of interest is from June 1st to August 31, therefore to compute a drought metric for June 1st, one will need a complete data record back to May 3rd (30 days including June 1st) for each year included in the analysis. Thus, for a 30-day timescale, completeness of record was evaluated from May 1st to August 31st. Alternatively, for a 90 day timescale, completeness is required to March 4th, thus, completeness of record was evaluated from March 1st to August 31st. Therefore, differences in timescale cause differences in the number of stations ultimately evaluated for additional analysis. All analysis was conducted in the R programming environment^38^. We accessed GHCN data using the RNOAA package^39^.

### Statistical analysis

#### Moving window operations

Moving window operations are at the core of the analysis presented in this study. Each computation is conducted without the use of data from the future with respect to the time period of interest. For example, any operation conducted in 2015 would only consider data in 2015 and prior. This is important in order to emulate the analysis that would have been conducted in 2015 (conventional drought monitoring does not use future projections in analysis). Although, it is important to acknowledge that it is common practice to retrospectively compute drought metrics (or anomalies) based on a reference period, that includes data that is in the future from a period of interest.

#### Gamma probability distribution estimation

Fitting probabilistic distributions to empirical data is one of the most important steps in computing drought metrics. Estimated distributions contextualize conditions with respect to expected outcomes in order to normalize data into anomalies. In this study, we fit a gamma probability distribution to an aggregated (summed) precipitation time series. To do so, raw daily precipitation time series were first aggregated over three timescales, 30, 60 and 90 days, to compute the annual summed precipitation time series for a day of interest (for example, June 1st). Once an aggregated precipitation time series was computed for a given site and day, we computed the unbiased sample probability-weighted moments of the aggregated precipitation data, which were then converted to L-moments (linear combination of traditional moments). Estimates of the gamma distribution parameters (rate and shape) were then computed given the L-moments of the data. This procedure was conducted using the lmomco package^40^.

#### Standardized precipitation index computation

The Standardized Precipitation (SPI) was designed by McKee and others^15^ to standardize precipitation time series across a record in order to normalize precipitation anomalies in both time and space while still accounting for non-normal distributions. To calculate the SPI, we computed the cumulative distribution function (CDF) of the aggregated precipitation time series using the associated gamma distribution fit (described above). The CDF values were then evaluated within an inverse Gaussian function with a mean of zero and a standard deviation of one to obtain the final SPI value. This “normalization” of the data centers CDF values of 0.5 (average timescale summed precipitation) about an SPI value of zero. Wet[dry] conditions have CDF values > [<] 0.5 and SPI values > [<] 0.

#### Drought metric bias

To compute the average difference (bias) between climatologies of differing lengths, we computed the median difference between daily summer time (June 1st—August 31st) SPI values for a long and short period of record. Our two time periods of record were: 1. The “period-of-record’ climatology which is composed of at least 70 years of complete data and 2. The most current 30-year record (“contemporary” record) which was composed of at least 25 years of complete data within the 30-year moving window (i.e. for 2020, 1991–2020, or for 2019, 1990–2019, and so on). The final bias was then computed as the median daily period-of-record SPI value minus the contemporary SPI value. Negative [positive] values represent locations where the period-of-record SPI value is on average lesser [greater] then the contemporary SPI value (dry [wet] bias). We computed the average bias for all observations in the study (Fig. S2). To evaluate if bias varied as a function of wetness/dryness state, we also computed the bias for breaks in the period-of-record SPI timeseries. Breaks were defined as period-of-record SPI > 2, > 2 SPI > 1, 1 > SPI > −1, −1 > SPI > −2 and −2 > SPI (from wet to dry, respectively, Fig. 5, S3, S4, S5, S6).

To aid in visually evaluating spatial patterns in drought metric bias we computed krigged maps of bias for all observations together and for each of the wetness/dryness categories separately. To generate these krigged maps we fit a variogram to each of the datasets using the automap^41^ and gstat^42^ packages in the R programming environment. Once a variogram was fit to the data we predicted the krigged surface of bias using the gstat package across a 1/3° x 1/3° raster in the WGS84 (EPSG 4326) coordinate projection system.

#### Monte Carlo analysis

In order to evaluate the absolute SPI error associated with parameterizing the gamma probability distribution with differing climatology lengths we ran three separate Monte Carlo simulations. The three simulations were meant to capture a range of scenarios under which SPI may be computed. The three simulations were: (1) simulation of a single stationary distribution with known gamma distribution parameters, (2) simulation of many stationary distributions with known gamma distribution parameters (parameter pairs sampled from the observed distribution of gamma parameters, Fig. S1), and (3) Simulation of a non-stationary distribution with known gamma distribution parameters that vary through time (parameter pair time series derived from GHCN site data, for example, Fig. 2). For all simulations, CDF and SPI error was assessed based on the most contemporary observation (“today’s” value). This methodology most closely mimics real-time drought monitoring processes.

#### Stationary distribution Monte Carlo analysis (single parameter pair)

To evaluate how the absolute SPI error varied as a function of the number of observations (years) in each climatology, we conducted an iterative experiment (Monte Carlo simulation). We defined a rate and shape parameter pair from which we generated random samples. The random samples come from a known distribution, thus we computed the true CDF and SPI values associated with that random sample. Probabilistic CDF and SPI values were computed based on fitting a gamma distribution to the randomly generated data of differing lengths, from 1 to 100 samples in 1 sample increments. The associated gamma distribution parameters were also stored to evaluate the convergence of estimated parameters towards the known gamma distribution parameters for differing climatology lengths. The absolute SPI and CDF error were computed by subtracting the probabilistic value from the true value for the most current observation (synonymous with the 2020 value used in operational drought monitoring). We repeated this process 1000 times, generating new data for each simulation. Finally, we summarized the results of the 1000 simulations by computing the median and interquartile range (IQR) of the gamma distribution parameters as well as the absolute CDF and SPI error for each climatology length (Fig. 3a).

#### Stationary distribution Monte Carlo analysis (multiple parameter pairs)

Following the method described above, but focusing on the absolute SPI error, we replicated the Monte Carlo simulation using 100 randomly sampled parameter pairs based on the full parameter space of observed parameters captured in this study (Fig. S1, white scatter points; June 1–August 31; 1991–2020, 30-, 60- and 90-day timescales, parameter space *n* = 4,907,001). This Monte Carlo simulation was meant to evaluate if the error estimation described above is dependent on the gamma distribution parameter pair evaluated. For example, it is possible that distributions with greater variability may require more samples to adequately fit the probability distribution when compared to a distribution with lesser variability. Thus, we replicated the analysis above with 1000 simulations per parameter pair. In the same manner as above, we computed the median and IQR absolute SPI error for each individual parameter pair and for all simulations and parameters together (Fig. 3b).

#### Non-stationary distribution Monte Carlo analysis

To evaluate the effect of a non-stationary distribution on the absolute SPI error, we adapted the method described above using parameter pairs that are dynamic in time. Therefore, for each of the climatology lengths (1−100 samples), random data was generated from a new distribution. The distributional parameter pairs used in this simulation were derived from the 30-year moving window analysis of observed data at 11 GHCN sites (Fig. 4). These 11 sites represent locations with 100 years or more of complete precipitation records (as defined above) for a given timescale. Therefore, the annual changes in parameter values in the simulation are representative of true, observed distributional shifts (for example, Fig. 2; Fig S7; Fig S8). In order to simulate annual shifts in the gamma distribution, we infilled any missing rate and shape parameters using a spline function (shown in Fig. 2). However this was only done if there were missing values (<2% of data), thus we used the real 30-year moving window parameter pairs whenever possible.

First, we used the gamma distribution pairs from GHCN site USC00381770, located at Clemson University, South Carolina (Fig. 2). For each simulation, we generated a random sample from the time-specific gamma distribution for each of the climatology length values (1–100). Probabilistic SPI values were computed by fitting a gamma distribution to the randomly generated samples from each of the generative distributions. Therefore, at a climatology length of 30 observations (years), the probabilistic value was computed by fitting a gamma distribution to the randomly generated 30 observations from the 30 generative distributions (replicating an analysis from 1991 to 2020). Similarly, for a climatology length of 90 observations, the probabilistic gamma distribution was fit to the data from the 90 previous generative distributions (effectively 1931 to 2020). The absolute SPI error for the most recent observation (effectively 2020) was computed by subtracting the probabilistic value from the true value computed using the most current gamma distribution (2020). This analysis was conducted for climatology lengths from 1 to 100 and the simulation was conducted 1000 times. These results were summarized by computing the median and IQR of the absolute SPI error (Fig. 4a). We replicated this analysis for 10 additional GHCN sites and for the 30-, 60- and 90-day timescales (Fig. 4b).

## Supplementary information

Supplementary Information

## Data Availability

All data used in this analysis is publicly available and freely accessible using the code provided; however, derivative data generated in this study have been deposited in the Zenodo database under accession code 5047800.

## Code availability

All code used to perform this analysis can be accessed at https://github.com/zhoylman/drought-year-sensitivity.
