# Supplementary material for: Drought assessment has been outpaced by climate change: empirical arguments for a paradigm shift
Source: Nat Commun. 2022 May 17;13:2715. doi: 10.1038/s41467-022-30316-5 (PMC9114319; doi:10.1038/s41467-022-30316-5)
Supplement: Supplementary file 1 — Supplementary Information [file 41467_2022_30316_MOESM1_ESM.pdf]

Supplemental Material

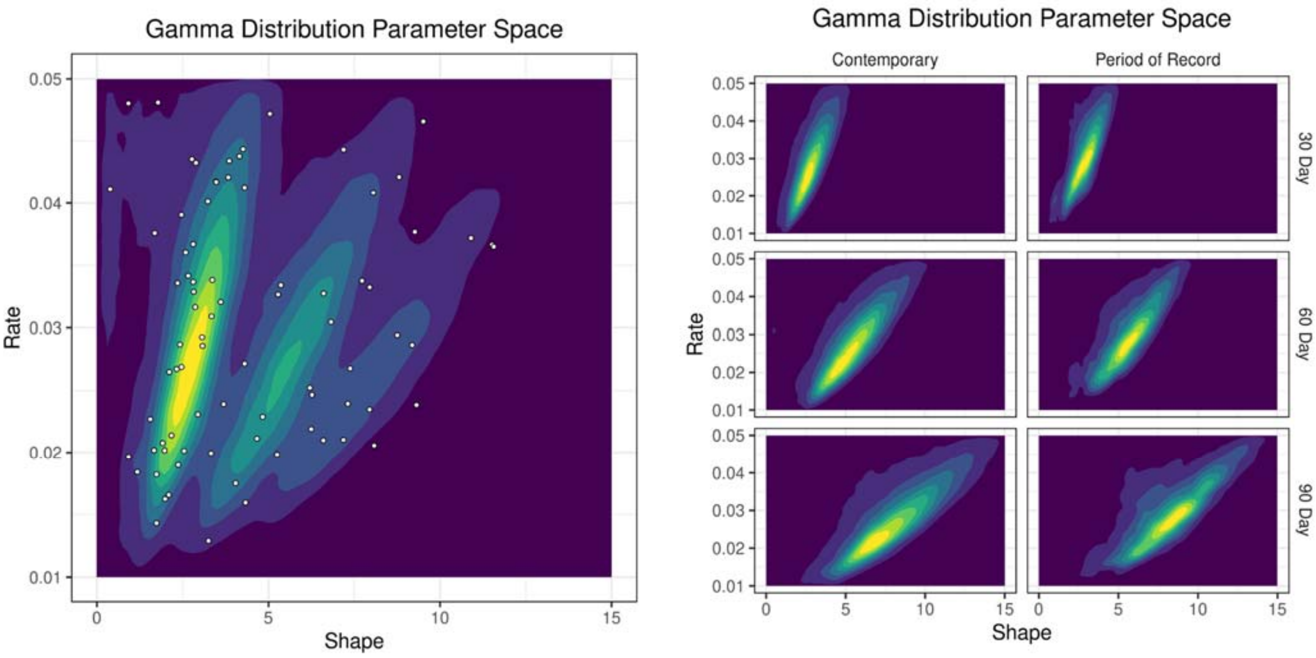

Figure S1: Observed gamma distribution parameter space for all time periods, timescales and Global Historical Climatology Network (GHCN) sites considered in this analysis.

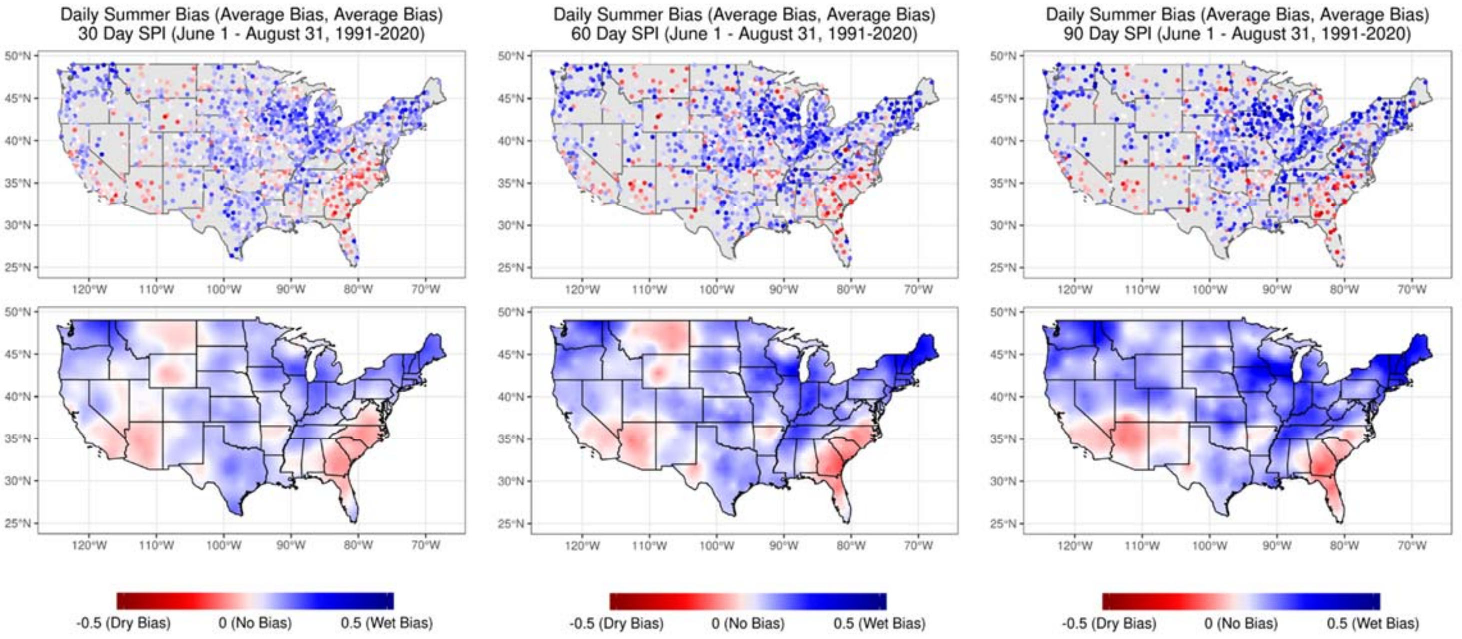

Figure S2: Average drought metric bias for the Standardized Precipitation Index (SPI) for Global Historical Climatology Network (GHCN) sites across the United States for all time periods considered. Bias was computed as the median daily difference between the period-of-record SPI and the 30 year ("contemporary") SPI from June 1 to August 31, 1991 – 2020. Dry bias (represented by red) denotes locations where the period-of-record reports conditions that are drier than the most recent 30 years for [left] 30 day, [middle] 60 day and [right] 90 day timescales.

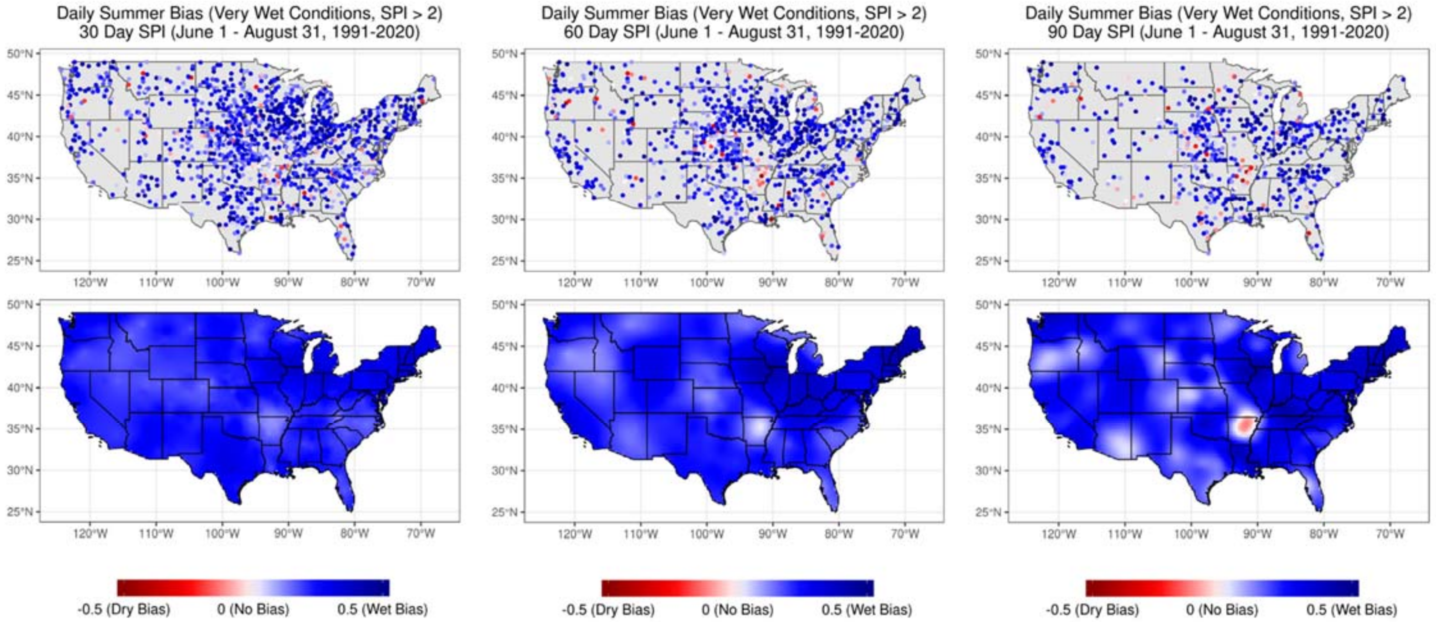

Figure S3: Drought metric bias for the Standardized Precipitation Index (SPI) for Global Historical Climatology Network (GHCN) sites across the United States during periods with SPI > 2 (very wet conditions) defined using the full period-of-record SPI timeseries. Bias was computed as the median daily difference between the period-of-record SPI and the 30 year ("contemporary") SPI from June 1 to August 31, 1991 – 2020. Wet bias (represented by blue) denotes locations where the longest period-of-record reports conditions that are wetter than the most recent 30 year climatology for [left] 30 day, [middle] 60 day and [right] 90 day timescales.

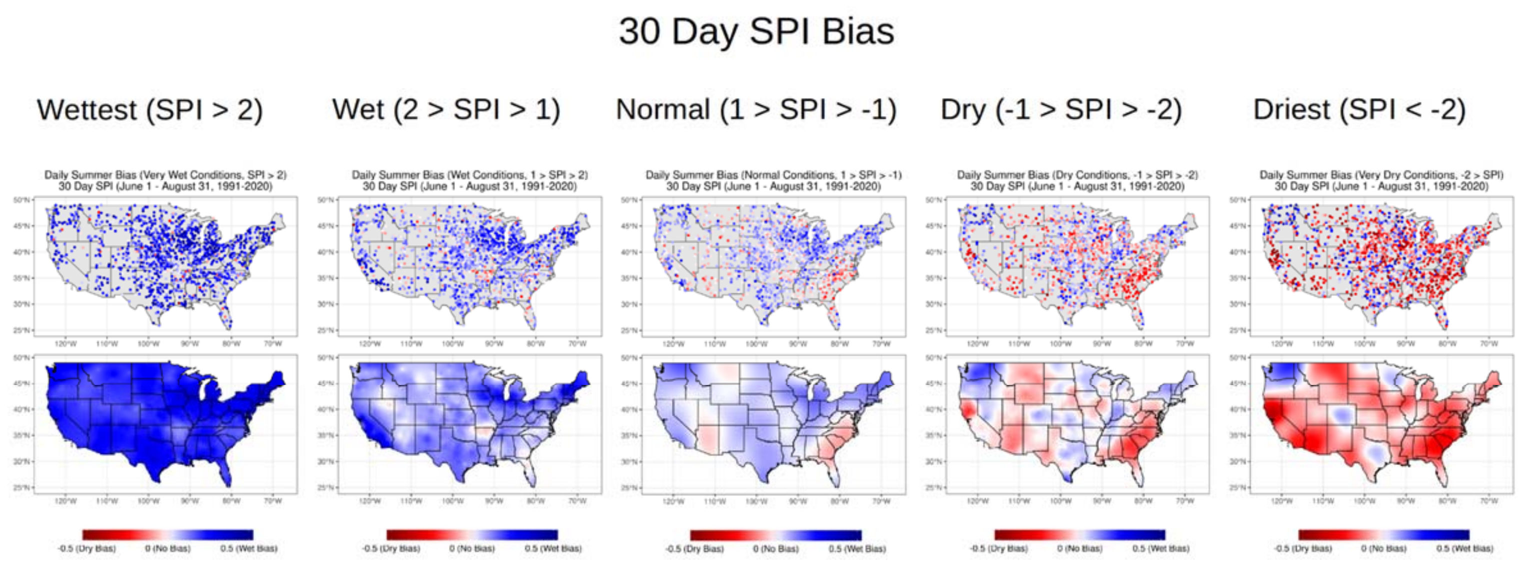

Figure S4: Drought metric bias for the Standardized Precipitation Index (SPI) computed using a 30 day timescale for Global Historical Climatology Network (GHCN) sites across the United States aggregated by dryness defined using the period-of-record SPI timeseries. Bias was computed as the median daily difference between the period-of-record SPI and the 30 year ("contemporary") SPI from June 1 to August 31, 1991 – 2020. Dry[wet] bias (represented by red[blue]) denotes locations where the period-of-record reports conditions that are drier[wetter] than the past 30 years for wet to dry conditions, from left to right respectively.

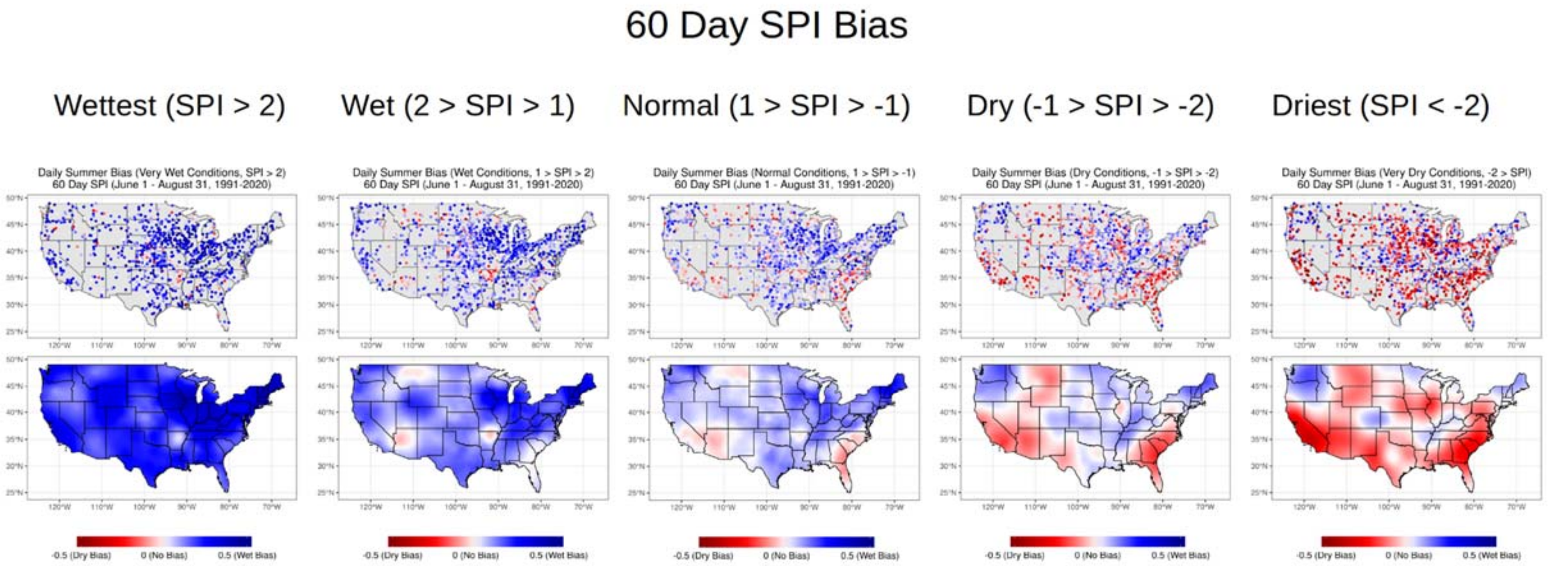

Figure S5: Drought metric bias for the Standardized Precipitation Index (SPI) computed using a 60 day timescale for Global Historical Climatology Network (GHCN) sites across the United States aggregated by dryness defined using the period-of-record SPI timeseries. Bias was computed as the median daily difference between the period-of-record SPI and the 30 year (“contemporary”) SPI from June 1 to August 31, 1991 – 2020. Dry[wet] bias (represented by red[blue]) denotes locations where the period-of-record reports conditions that are drier[wetter] than the past 30 years for wet to dry conditions, from left to right respectively.

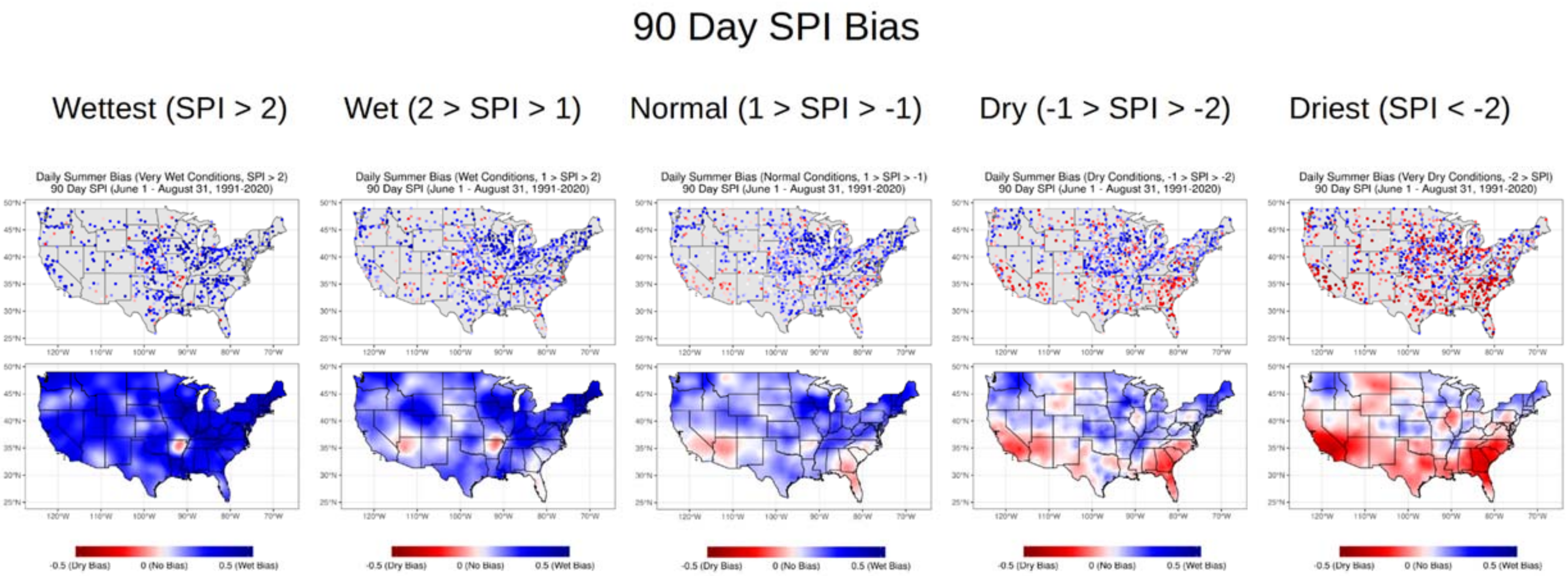

Figure S6: Drought metric bias for the Standardized Precipitation Index (SPI) computed using a 90 day timescale for Global Historical Climatology Network (GHCN) sites across the United States aggregated by dryness defined using the period-of-record SPI timeseries. Bias was computed as the median daily difference between the period-of-record SPI and the 30 year ("contemporary") SPI from June 1 to August 31, 1991 – 2020. Dry[wet] bias (represented by red[blue]) denotes locations where the period-of-record reports conditions that are drier[wetter] than the past 30 years for wet to dry conditions, from left to right respectively.

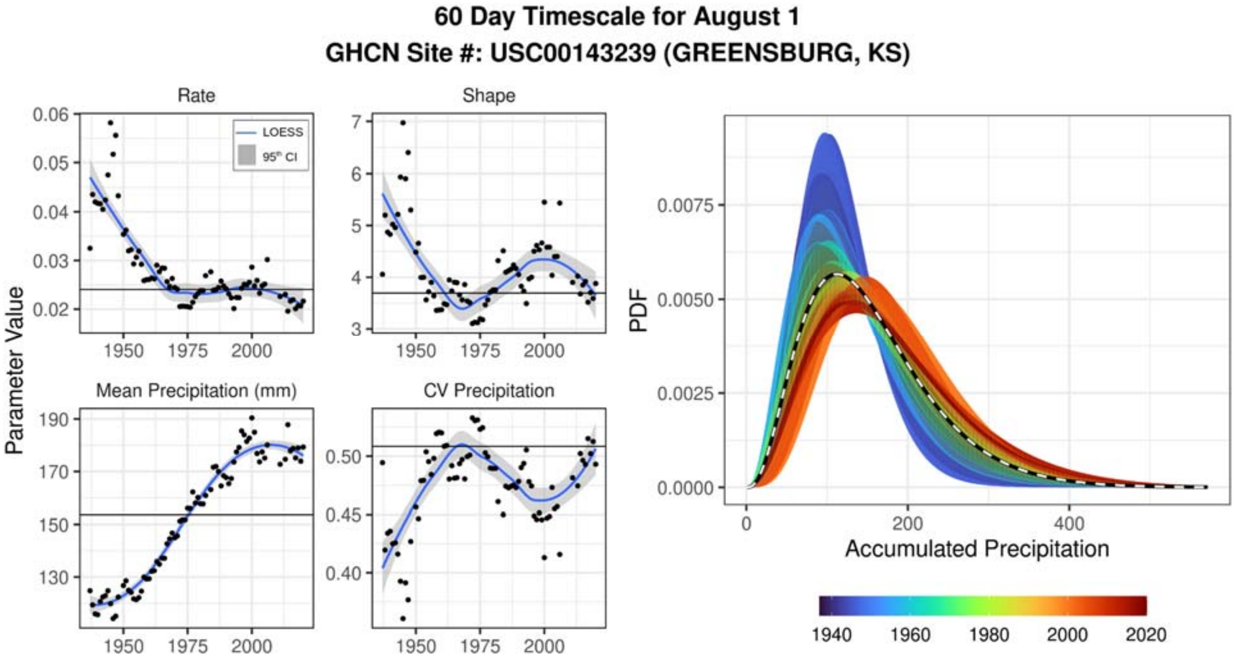

Figure S7: Probability distribution shift for Global Historical Climatology Network (GHCN) site USC00143239 located at Greensburg in Kansas. [left] Subplots show 30 year moving window values of the rate and shape gamma distribution parameters, mean precipitation and coefficient of variation (CV) of precipitation for a 60 day time scale on August 1st. Horizontal lines represent values computed using the temporally integrated distribution. [right] Probability density functions (PDFs) for each of the 30 year moving window. The color scale represents the 30 year moving window's final year and the black and white dashed line represents the temporally integrated PDF.

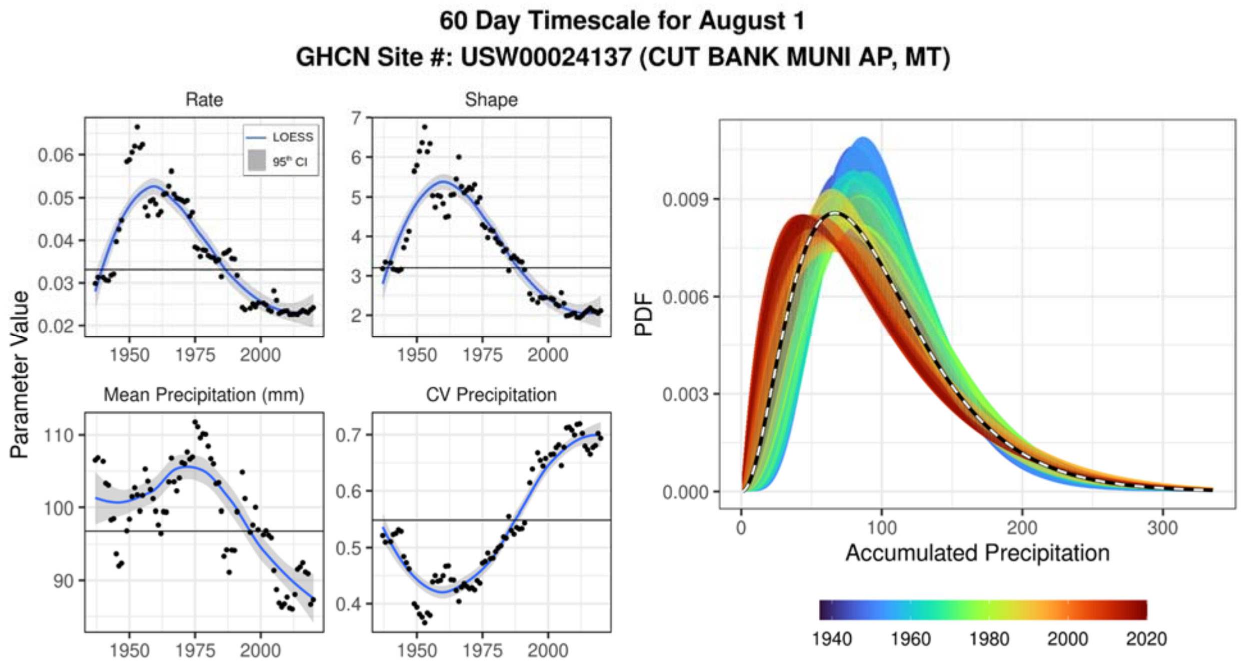

Figure S8: Probability distribution shift for Global Historical Climatology Network (GHCN) site USW00024137 located at Cut Bank Municipal Airport in Montana. [left] Subplots show 30 year moving window values of the rate and shape gamma distribution parameters, mean precipitation and coefficient of variation (CV) of precipitation for a 60 day time scale on August 1st. Horizontal lines represent values computed using the temporally integrated distribution. [right] Probability density functions (PDFs) for each of the 30 year moving window. The color scale represents the 30 year moving window's final year and the black and white dashed line represents the temporally integrated PDF.
